# Beyond Asbestos: Malignant Pleural Mesothelioma Revealing Lynch Syndrome Through Mismatch Repair Deficiency

**DOI:** 10.7759/cureus.101550

**Published:** 2026-01-14

**Authors:** Stefanni Vivanco, Sebastian Vega, Angello Muguruza, Andrea Garzón, Sergio Daniel Zabaleta Orozco, Michael Hernández

**Affiliations:** 1 Medical Department, Universidad Peruana Cayetano Heredia, Lima, PER; 2 Medical Department, Universidad Ricardo Palma, Lima, PER; 3 Medical Department, Universidad Central del Ecuador, Quito, ECU; 4 Medical Department, Universidad Surcolombiana, Neiva, COL; 5 Medical Department, Universidad de Ciencias Médicas de Pinar del Río, Pinar del Río, CUB

**Keywords:** asbestos exposure, dna mismatch repair deficiency, hereditary cancer, lynch syndrome, mesothelioma

## Abstract

Lynch syndrome (LS) is a hereditary cancer predisposition condition. We report a rare case of malignant pleural mesothelioma occurring in a patient with this syndrome. The interplay between genetic susceptibility and environmental exposure remains underrecognized. We describe a case of a patient with multiple primary malignancies, including mesothelioma. A 70-year-old woman, with a history of endometrial, breast, and colorectal cancers, developed malignant pleural mesothelioma following 15 years of residence in a mining area with probable asbestos exposure. Immunohistochemistry revealed a loss of PMS2 expression, supporting a defect in MMR. This report documents an exceptional coexistence of malignant pleural mesothelioma and LS, suggesting that impaired DNA repair may lower the carcinogenic threshold for asbestos exposure. Clinicians should consider LS in patients with multiple or atypical malignancies, including mesothelioma, and recommend comprehensive MMR testing and genetic counseling for at-risk relatives.

## Introduction

Lynch syndrome (LS), or hereditary non-polyposis colorectal cancer (HNPCC), is the most common cause of hereditary colorectal cancer and is associated with germline mutations in mismatch repair (MMR) genes (MLH1, MSH2, MSH6, PMS2, and EPCAM) [1,2]. It accounts for 2-4% of all CRC cases and confers a lifetime risk of 52-82% for developing the disease [3-5]. In addition, LS is linked to several extracolonic tumors, including those of the endometrium, ovary, skin, and, less frequently, the pleura [6-9].

The estimated incidence of LS is 1 in 300 individuals, likely underestimated due to limited systematic genetic screening [10]. Its clinical relevance lies in the predisposition to multiple primary neoplasms, which requires intensive surveillance [11-13].

Malignant pleural mesothelioma is a rare neoplasm; most of the approximately 30,870 global cases correspond to this location [13]. Although typically associated with asbestos exposure, cases linked to germline mutations in MMR genes, particularly MSH2, have also been reported [9,14]. This association complicates the differential diagnosis with pleural metastases and expands the recognized tumor spectrum of LS.

The present case is notable for the coexistence of multiple neoplasms: CRC, breast cancer, endometrial cancer, and pleural mesothelioma in the same patient, representing an unusual pattern within LS and highlighting the importance of recognizing atypical manifestations and performing genetic evaluation in families with multiple oncologic histories.

The aim of this report is to describe an atypical presentation of LS with pleural involvement, emphasizing the diagnostic and molecular implications and the need for expanded surveillance in high-risk families.

## Case presentation

A 70-year-old woman with a history of endometrial cancer treated 20 years ago with radical hysterectomy, chemotherapy, and brachytherapy; right breast cancer operated on 10 years ago; and colon cancer 9 years ago treated surgically without systemic therapy due to an enterocutaneous fistula. She reported a strong family history of malignancies suggestive of a hereditary cancer syndrome. Among her siblings, one had two episodes of colorectal cancer and cutaneous squamous cell carcinoma, while two other siblings were each diagnosed with a single episode of colorectal cancer. In the second generation, one nephew had three episodes of colorectal cancer. Additionally, one niece was diagnosed with two episodes of colorectal cancer and endometrial cancer, and another niece had two episodes of colorectal cancer and biliary tract cancer.

She lived for 15 years in a mining area in Pasco, with chronic exposure to mining dust from blasting; asbestos exposure was suspected but not directly measured/confirmed.

During an outpatient follow-up, a non-contrast thoracic-abdominal-pelvic computed tomography (CT) scan identified subpleural pseudonodules smaller than 10 mm in the right lower lobe and a left laminar pleural effusion. A subsequent positron emission tomography-computed tomography (PET-CT) showed a right hilar mediastinal lymph node measuring 0.6 × 0.6 cm, suspicious for metastatic activity, along with bilateral laminar pleural effusion.

Video-assisted thoracoscopic surgery (VATS) was performed with resection of pulmonary nodules, mediastinal lymph nodes, and pleural biopsies (pleural and diaphragmatic). During the procedure, pleural carcinomatosis (visceral, parietal, diaphragmatic, and pericardial pleura), multiple mediastinal lymphadenopathies, and multiple pulmonary nodules were observed. Up to that point, she had only presented right-sided chest pain, without dyspnea or any other symptoms.

The mediastinal biopsy revealed soft tissue with mucoid material and isolated atypical epithelioid cells. The incisional lung biopsy showed pulmonary parenchyma and pleural surface with nodules of mucoid material and neoplastic proliferation of malignant epithelioid cells. The pleural biopsy demonstrated fibromuscular tissue with neoplastic proliferation of malignant epithelioid cells in a mucoid background.

**Figure 1:**
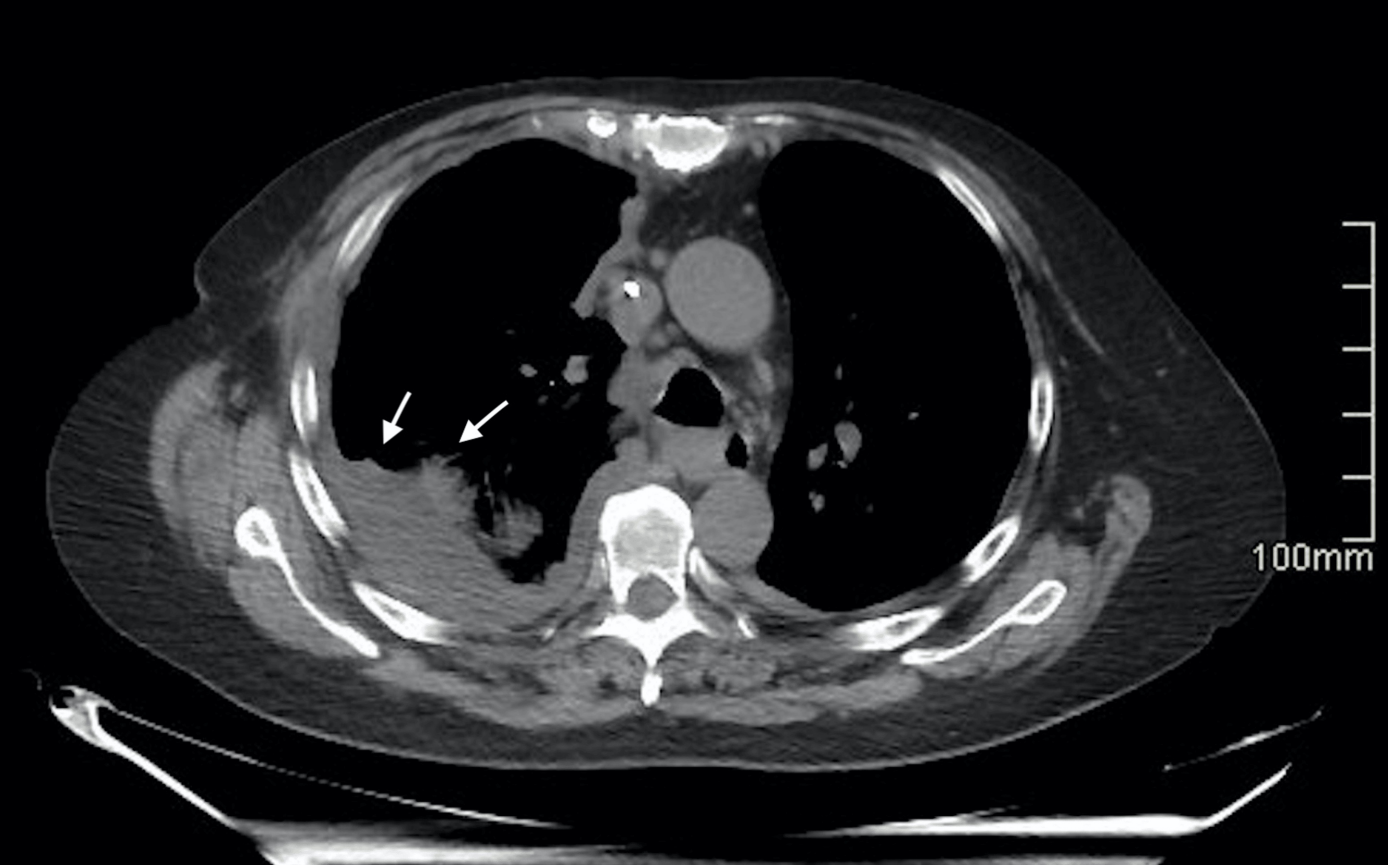
A non-contrast thoracic CT scan identified diffuse tumoral thickening of the pleura in the right hemithorax

**Figure 2:**
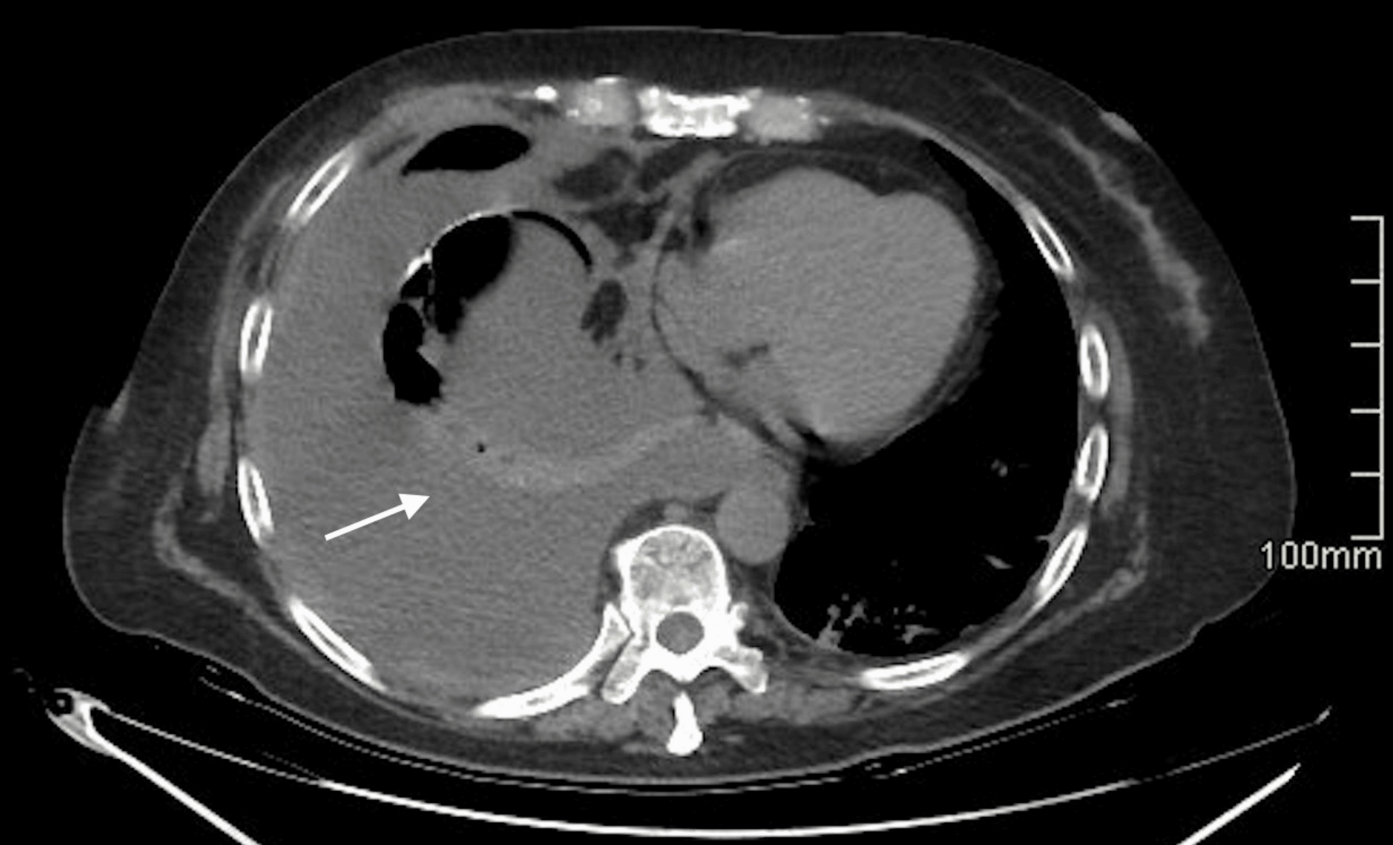
A non-contrast thoracic CT scan identified moderate right pleural effusion

Histopathological examination of the pleural biopsy revealed features consistent with epithelioid malignant mesothelioma. Immunohistochemical analysis of the tumor cells demonstrated positivity for CK7, WT1, and D2-40, with negativity for CK20, GATA3, PAX8, CDX2, TTF1, ER, SOX10, calretinin, and CD68, supporting the diagnosis of epithelioid malignant mesothelioma with areas of rhabdoid pattern. Given the patient’s personal oncologic history and a strong family history suggestive of a hereditary cancer syndrome, additional mismatch repair (MMR) immunohistochemistry was subsequently performed. This analysis demonstrated isolated loss of nuclear PMS2 expression, with preservation of MLH1, MSH2, and MSH6, consistent with mismatch repair deficiency and raising strong suspicion for Lynch syndrome. The diagnosis was established based on histopathologic evaluation and immunohistochemistry at the treating institution; an external confirmatory pathology review was not performed. Pleural thickening with nodules and bilateral pleural effusions were identified, as shown in Figures 1, 2.

The patient was treated with multiple thoracic drainages and chemotherapy. She received immunotherapy with nivolumab and ipilimumab. Notably, a first-degree relative (her brother) was later diagnosed with colorectal cancer and a cutaneous squamous cell carcinoma, both subsequently linked to Lynch syndrome, further supporting the familial nature of this condition.

## Discussion

This case highlights the complexity of Lynch syndrome (LS), particularly when malignancies fall outside the classic tumor spectrum. The presentation of malignant pleural mesothelioma in a patient with confirmed LS-associated cancers, combined with similar malignancies identified in her brother, illustrates the interplay between genetic predisposition and environmental exposure in carcinogenesis. This case, therefore, provides an opportunity to examine how LS predisposition may modulate tumor behavior in the presence of environmental carcinogens.

Malignant mesothelioma is an aggressive neoplasm strongly linked to asbestos exposure, most commonly in occupational settings. Although globally rare [13], prolonged or repeated exposure to asbestos remains a well-established risk factor [15]. In this case, the development of mesothelioma introduces the critical environmental component. A large case-control study has demonstrated that asbestos exposure significantly increases the risk of malignant pleural mesothelioma, even in individuals without direct occupational contact [15]. This supports the evidence that low-level or para-occupational exposure can still be carcinogenic.

The patient’s long-term residence (15 years) in a mining zone with dust generated from blasting represents a plausible source of environmental asbestos exposure. Additionally, an ecological study of mesothelioma mortality in Peru identified elevated rates in regions with high mineral fiber importation, including Pasco, where the patient resided [16]. The timeline is consistent with known disease biology: mesothelioma typically manifests decades after initial exposure, aligning with the 15-year latency observed here [17].

The familial history further indicates LS as the underlying genetic disorder. Multiple first-degree relatives, including the patient’s brother, were diagnosed with colorectal and cutaneous malignancies associated with LS. The coexistence of endometrial, breast, colorectal cancer, and now malignant mesothelioma in the index patient underscores the broad phenotypic variability reported in LS [13,18].

Diagnosing malignant mesothelioma in this patient presented notable challenges. Notably, the tumor was negative for calretinin, an unusual finding given that approximately 90% of epithelioid malignant mesotheliomas express this marker [19]. However, rare calretinin-negative cases have been reported, underscoring the importance of using a broad immunohistochemical panel. The final diagnosis of epithelioid malignant mesothelioma was supported by characteristic histological features, including epithelioid morphology with rhabdoid areas. Immunohistochemical analysis demonstrated tumor cell positivity for WT1, D2-40, and CK7, with negativity for markers associated with alternative primary malignancies, including CK20, GATA3, PAX8, CDX2, TTF1, ER, SOX10, calretinin, and CD68, thereby excluding other potential primary sites.

The coexistence of mesothelioma, MMR deficiency, and environmental asbestos exposure suggests a synergistic carcinogenic effect. Shih et al. reported only two cases of LS-associated mesothelioma, both occupationally exposed [20], illustrating the rarity of this association. Our case strengthens the limited evidence by proposing a plausible mechanism: defective DNA mismatch repair may lower the carcinogenic threshold, allowing even modest or intermittent asbestos exposure to induce mesothelioma in genetically predisposed individuals.

## Conclusions

We describe a rare occurrence of malignant pleural mesothelioma in a patient with Lynch syndrome, broadening the tumor spectrum associated with mismatch-repair deficiency. The coexistence of MMR-deficient malignancy and environmental asbestos exposure suggests a potential synergistic carcinogenic effect. Clinicians should consider LS in patients with multiple or atypical tumors, and genetic counseling should be offered to at-risk relatives. Further studies are needed to clarify how MMR defects modify susceptibility to environmental carcinogens.

## References

[1] Zhu B, Liu M, Mu T (2024). Quadruple primary tumors in a lynch syndrome patient surviving more than 26 years with genetic analysis: a case report and literature review. Front Oncol.

[2] (2025). Instituto Nacional del Cáncer: Diccionario de cáncer del NCI. https://www.cancer.gov/espanol/publicaciones/diccionarios/diccionario-cancer/def/sindrome-de-lynch.

[3] Pallatt S, Nambidi S, Adhikary S (2025). A brief review of Lynch syndrome: understanding the dual cancer risk between endometrial and colorectal cancer. Oncol Rev.

[4] Lynch HT, de la Chapelle A (2003). Hereditary colorectal cancer. N Engl J Med.

[5] Khaddour K, Fields RC, Ansstas M, Rosman IS, Ansstas G (2020). Metachronous cutaneous squamous cell carcinoma in a young patient as the only presenting symptom to uncover Lynch syndrome with MLH1 Germline mutation. Hered Cancer Clin Pract.

[6] Mohiuddin M (2025). Hereditary nonpolyposis colon cancer (Lynch syndrome): an emerging public health concern. Health Sci Rep.

[7] Castro-Mujica M del C, Barletta-Carrillo C (2018). Síndrome de Lynch: aspectos genéticos, clínicos y diagnósticos. Rev Gastroenterol Perú.

[8] Dal Buono A, Puccini A, Franchellucci G (2024). Lynch syndrome: from multidisciplinary management to precision prevention. Cancers (Basel).

[9] Arulananda S, Thapa B, Walkiewicz M (2018). Mismatch repair protein defects and microsatellite instability in malignant pleural mesothelioma. J Thorac Oncol.

[10] Sherman S, Ojha S, Menon G, Laslett N (2025). Hereditary Nonpolyposis Colon Cancer (Lynch Syndrome). https://www.ncbi.nlm.nih.gov/books/NBK564511/.

[11] Bhattacharya P, Leslie SW, McHugh TW (2025). Lynch Syndrome (Hereditary Nonpolyposis Colorectal Cancer). https://www.ncbi.nlm.nih.gov/books/NBK431096/.

[12] Peltomäki P, Nyström M, Mecklin JP, Seppälä TT (2023). Lynch syndrome genetics and clinical implications. Gastroenterology.

[13] Zhao Z, Li J, Tan F (2025). Assessing the global burden of mesothelioma: trends, socioeconomic influences, and asbestos exposure - a retrospective cohort study. Int J Surg.

[14] Kastrinos F, Balmaña J, Syngal S (2013). Prediction models in Lynch syndrome. Fam Cancer.

[15] Migliore E, Consonni D, Peters S (2022). Pleural mesothelioma risk by industry and occupation: results from the multicentre Italian study on the etiology of mesothelioma (MISEM). Environ Health.

[16] Torres-Roman JS, Gomez-Rubio V, Sanchez-Trujillo L (2020). Geographic study of mortality due to mesothelioma in Peru and its evolution. Cancer Epidemiol.

[17] (2025). MedScape: Mesothelioma. https://emedicine.medscape.com/article/280367-overview.

[18] Møller P (2020). The prospective Lynch syndrome database reports enable evidence-based personal precision health care. Hered Cancer Clin Pract.

[19] Li D, Wang B, Long H, Wen F (2015). Diagnostic accuracy of calretinin for malignant mesothelioma in serous effusions: a meta-analysis. Sci Rep.

[20] Shih AR, Kradin RL (2019). Malignant mesothelioma in Lynch syndrome: a report of two cases and a review of the literature. Am J Ind Med.

